# Ascites.—I

**Published:** 1909-06-12

**Authors:** 


### ASCITES.—I

Ascites is the term which is used to denote an accumulation of serous fluid in the general peritoneal cavity. The amount of fluid may vary from a few ounces to several gallons.

There cannot be any very great amount of ascitic fluid present if the abdominal wall is flat, still less if it is retracted. In cases of ascites there is nearly always abdominal distension, the latter being uniform and varying with the amount of fluid which is present. If the quantity is large and it has accumulated rapidly the abdomen presents a rounded, globular appearance, and the umbilicus is no longer a depression. If an acute case, the umbilical region may be the most prominent; if, however, a large quantity of fluid has taken a considerable time to accumulate, bulging of the flanks is a marked feature of the general distension, so that the transverse diameter appears to be greater than the antero-posterior. If, again, the quantity of fluid is not great, only a slight bulging of the flanks may be noticed.

It must, of course, be remembered, that the appearance of the abdomen depends a good deal on the position of the patient during the examination. The above description applies to a patient who is lying perfectly flat upon his back, for if he be inclined to one side more than to the other the most dependent part becomes the most prominent, as the fluid has gravitated to that side of the abdomen. If the patient is standing or sitting upright the hypogastric and iliac regions will bulge. In cases of some standing the skin of the abdominal wall becomes tense and shiny. With large effusions lineæ albicantes will develop in the lower half of the abdominal wall.

The umbilicus retains its position in the median line, and it remains nearer to the symphysis pubis than to the ensiform cartilage. In tuberculous peritonitis the skin in its immediate neighbourhood sometimes becomes reddened and œdematous, and there may be a purulent or even fæcal discharge from it. In cirrhosis of the liver it is often stated in text-books that the veins around the umbilicus may be dilated and tortuous, forming the so-called "caput medusæ," but in practice this occurrence is one of the very greatest rarity.

The superficial veins all over the abdomen and lower part of the chest may be dilated; if the blood within them is flowing in an upward direction it indicates obstruction to the inferior vena cava either by the weight of the ascitic fluid itself or else by the cause of the ascites—new growth, for instance—with the result that blood from the abdomen is compelled to seek the right auricle by the azygos veins or by the superior vena cava. The thighs, legs, and loins may be œdematous. The epigastric angle will very likely be widened out, and in severe cases the lower ribs may be everted.

Abdominal movements are restricted or even entirely suppressed, and respiration becomes thoracic in character. The cardiac impulse may be displaced upwards or outwards, or both.

The abdominal wall is not necessarily tense. If the fluid is small in amount, or even if, though abundant, it has been a long time in accumulating, or has begun to decrease after being still more abundant, the parietes may be quite flaccid. If, on the other hand, the quantity of fluid is very large, or if it has collected rapidly, the abdominal wall may be so tense as to preclude palpation of the organs behind.

A fluid thrill is nearly always to be obtained by placing the palm of one hand in contact with the flank on one side and then flicking the other flank with the fingers of the other hand; a distinct wave or sense of vibration can be felt. In order to eliminate the thrill which is apt to be transmitted along the abdominal wall the patient or an assistant should place the side of his hand on the edge of a thin book or some similar object along the front of the abdomen vertically in the middle line. If this precaution is taken and a thrill is still obtained, fluid is certainly present, although it is not necessarily ascitic.

If the liver or spleen is enlarged, a layer of fluid is generally present between the anterior surface of the organ and the abdominal wall. If under these circumstances the tips of the fingers are placed upon the abdomen in the right or left hypochondriac region as the case may be, and are suddenly depressed, the fluid is displaced and the surface of the underlying enlarged organ is felt. This form of examination is called the "method of displacement" or "dipping," and it is one of the pathognomonic signs of the presence of free fluid in the peritoneal cavity.

The direction of the blood current in any dilated veins, the position of the cardiac impulse, the diminished respiration movements, and the condition of the umbilicus, mentioned above under the heading of inspection, can be checked by palpation. In malignant disease that has extended to the peritoneum the umbilicus may be felt fixed and thickened; infiltration of the remains of the urachus below the umbilicus may also be sometimes palpated.

The abdomen should always be examined with great care after the performance of paracentesis abdominis, for tumours and enlargements of organs may then be discovered which were previously hidden or obscured by the tenseness of the abdominal wall.

When the patient lies flat on his back the fluid gravitates to the posterior parts and sides of the peritoneal cavity, and the air-containing viscera float upwards and forwards, so that the percussion note in front is resonant, whereas in the flanks it is dull. As the fluid increases in quantity the line of dulness creeps forwards until it reaches from side to side across the middle line just above the pubes first, and thence steadily upwards; the line of junction between resonance above and dulness below is a curve whose convexity is downward. In extreme cases the abdomen may be dull all over, a condition which is not uncommon in children suffering from ascites, and which is also present in some cases of tuberculous peritonitis when the mesentery is thickened and shortened to such a degree that the intestines, being thus tied to the posterior abdominal wall, are unable to float forward. In a few such cases the bowels lie in the flanks and the fluid in front of them, giving rise to the physical signs which are usually regarded as typical of pregnancy or ovarian cyst—dulness in front and resonance in either flank.

*(To be continued.)*

